# Mild sleep restriction increases endothelial oxidative stress in female persons

**DOI:** 10.1038/s41598-023-42758-y

**Published:** 2023-09-16

**Authors:** Riddhi Shah, Vikash Kumar Shah, Memet Emin, Su Gao, Rosemary V. Sampogna, Brooke Aggarwal, Audrey Chang, Marie-Pierre St-Onge, Vikas Malik, Jianlong Wang, Ying Wei, Sanja Jelic

**Affiliations:** 1 Division of Pulmonary, Allergy, and Critical Care Medicine, Columbia University Vagelos College of Physicians and Surgeons, New York, NY USA; 2 Division of Nephrology, Columbia University Vagelos College of Physicians and Surgeons, New York, NY USA; 3 Division of Cardiology, Columbia University Vagelos College of Physicians and Surgeons, New York, NY USA; 4 NewYork-Presbyterian Morgan Stanley Children’s Hospital, Columbia University Vagelos College of Physicians and Surgeons, New York, NY USA; 5 Division of General Medicine, Columbia University Vagelos College of Physicians and Surgeons, New York, NY USA; 6 Columbia Center for Human Development and Columbia Stem Cell Initiative, Columbia University Vagelos College of Physicians and Surgeons, New York, NY USA; 7 Division of Biostatistics, Columbia University Vagelos College of Physicians and Surgeons, New York, NY USA

**Keywords:** Systems biology, Biomarkers, Cardiology, Diseases, Neurology

## Abstract

Sleep restriction is associated with increased cardiovascular risk, which is more pronounced in female than male persons. We reported recently first causal evidence that mild, prolonged sleep restriction mimicking “real-life” conditions impairs endothelial function, a key step in the development and progression of cardiovascular disease, in healthy female persons. However, the underlying mechanisms are unclear. In model organisms, sleep restriction increases oxidative stress and upregulates antioxidant response via induction of the antioxidant regulator nuclear factor (erythroid-derived 2)-like 2 (Nrf2). Here, we assessed directly endothelial cell oxidative stress and antioxidant responses in healthy female persons (n = 35) after 6 weeks of mild sleep restriction (1.5 h less than habitual sleep) using randomized crossover design. Sleep restriction markedly increased endothelial oxidative stress without upregulating antioxidant response. Using RNA-seq and a predicted protein–protein interaction database, we identified reduced expression of endothelial Defective in Cullin Neddylation-1 Domain Containing 3 (DCUN1D3), a protein that licenses Nrf2 antioxidant responses, as a mediator of impaired endothelial antioxidant response in sleep restriction. Thus, sleep restriction impairs clearance of endothelial oxidative stress that over time increases cardiovascular risk.

Trial Registration: NCT02835261

## Introduction

More than a third of US adults sleep less than recommended 7–8 h per night^1,2^. Insufficient sleep is associated with an increased cardiovascular risk, leading the American Heart Association to include sleep duration as the 8th metric of cardiovascular health in Life’s Essential 8^2–4^. Female persons report sleep disturbances more frequently and have a more pronounced inflammatory response and cardiovascular risk associated with insufficient sleep than males^2,4–8^. We recently reported that randomly allocated mild, prolonged sleep restriction causes endothelial inflammation and dysfunction, early steps in the development of cardiovascular disease, in healthy female persons^7^. However, the underlying mechanisms remain unclear.

One suggested major function of healthy sleep is prevention of oxidative stress, an important contributor to endothelial inflammation and dysfunction^9–11^. Insufficient sleep, much like other cardiovascular risk factors, including cigarette smoking, hyperlipidemia, hypertension, and diabetes, generates intracellular oxidative stress^11^. Studies in Drosophila and rodent models have shown that sleep restriction increases oxidative stress (defined as increased generation of reactive oxygen species) and upregulates antioxidant response via induction of the antioxidant regulator nuclear factor (erythroid-derived 2)-like 2 (Nrf2), a redox sensitive transcription factor that is kept in a latent state through its interaction with its repressor cullin-3 (Cul3)-containing ubiquitin ligase complex^12–14^. In response to increased oxidative stress, an adaptor protein Kelch-like ECH-associated protein 1 (Keap1) that binds to Nrf2 and Cul3 is modified and ubiquitin ligase complex is inactivated, allowing for Nrf2 accumulation and translocation into the nucleus where it binds to the antioxidant response element (ARE) and initiates the transcription of antioxidant genes^15^.

Organ-specific overexpression of antioxidant genes rescues the survival of severely sleep-deprived Drosophila^11^ and activation of the Nrf2-ARE pathway confers protection from cardiovascular diseases^16^, suggesting that intact antioxidant responses are essential to counteract detrimental effects of sleep restriction. Studies of the effects of insufficient sleep on oxidative stress in model organisms employed severe, acute sleep restriction or genetic manipulations that limit models’ lifespan^11,17^. Such extreme, short-term sleep curtailment has limited relevance to predominant populational sleep patterns of chronic, mild sleep curtailment owing to maintaining work/life balance in modern societies^2,4,11,17,18^. Whether chronic, mild sleep curtailment that mimics “real-life” sleep patterns affect endothelial oxidative stress and antioxidant responses is unknown. Using a randomized crossover design, we assessed oxidative stress and antioxidant responses directly in endothelial cells (ECs) freshly harvested from healthy female participants before and after objectively monitored 6 weeks of mild sleep restriction or adequate sleep.

## Results

### Sleep restriction increases endothelial oxidative stress

After completing a 2 week actigraphy screening to confirm that their habitual sleep duration is adequate (7–9 h daily), healthy female participants were randomized to a 6 week adequate sleep phase (sleep duration between regular bedtime and wake-time determined during actigraphy screening) or a 6 week mild sleep restriction phase (delaying bedtime by 1.5 h and keeping wake-time constant) followed by a 6 week washout period and crossover to the alternate sleep phase (Supplementary Fig. S1). Fifty-one participants were randomized to adequate sleep or sleep restriction. Out of 51 participants who underwent randomization, 16 did not complete at least one study phase and had no data available for analysis (13 failed to start any sleep phase and 3 did not complete the first phase). Three participants completed adequate sleep but not sleep restriction phase and 32 completed both phases of the study. Thus, 35 participants completed at least 1 phase and had data available for intention-to-treat analysis (n = 35; mean ± SD age 36 ± 14 years; body mass index [BMI] 25 ± 3 kg/m^2^; 49% racial minorities; baseline sleep duration 7 h 28 min ± 28 min). Mean reduction in sleep duration during the sleep restriction phase was 1 h 20 min/night compared with adequate sleep phase (mean ± SD 6 h 09 min ± 26 min vs. 7 h 28 min ± 20 min, *p* < 0.001). Sleep duration was monitored objectively by continuous actigraphy during both study phases.

**Figure 1:**
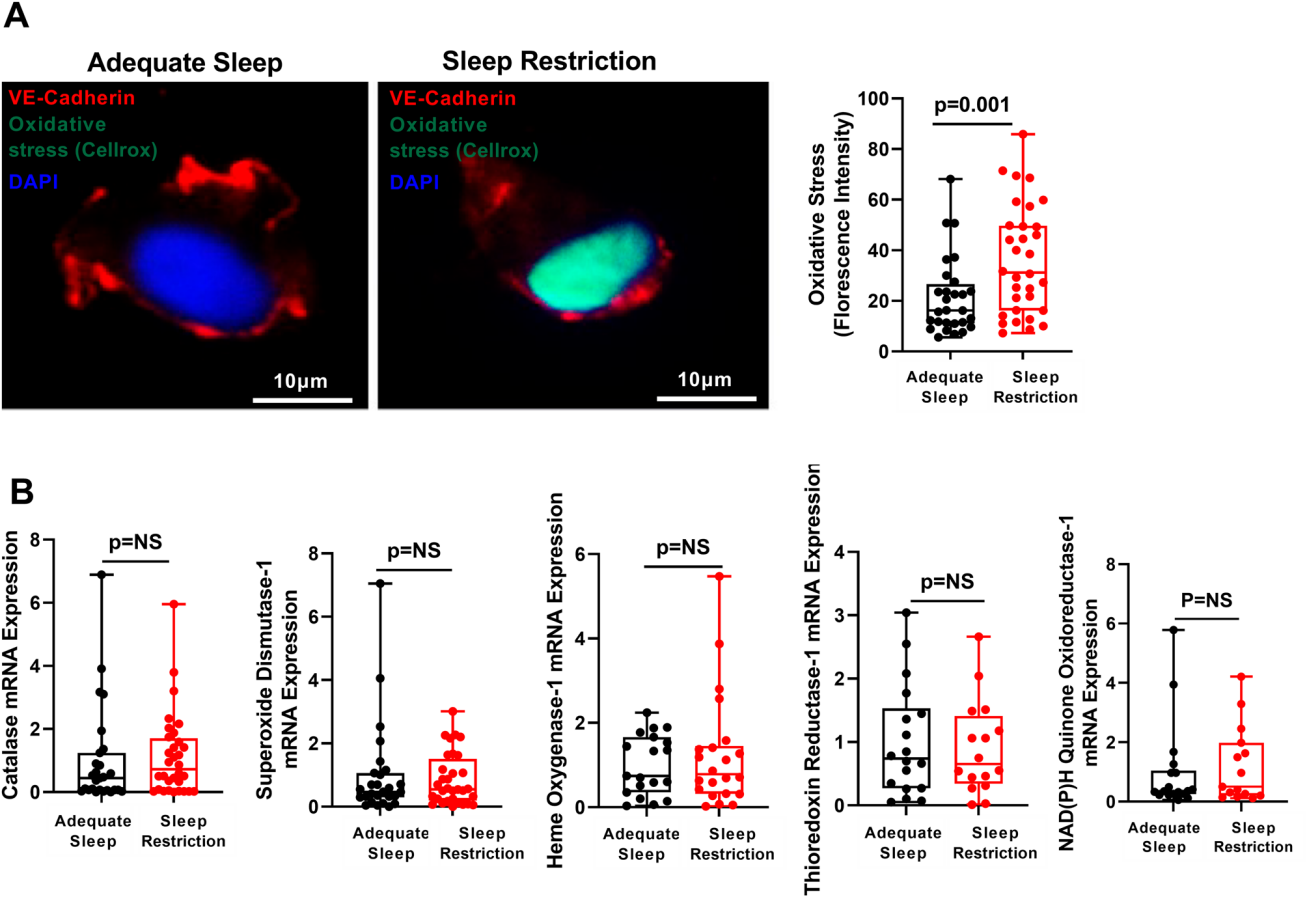
Sleep restriction increases endothelial oxidative stress without upregulating antioxidant responses. (**A**) Representative images and Box and Whisker Plot quantitating oxidative stress (nuclear fluorescence intensity of the fluorogenic probe activated by reactive oxygen species and subsequent binding to DNA) adjusted for baseline values in endothelial cells harvested from healthy participants after adequate sleep (n = 31) and after sleep restriction (n = 32). No pre-specified covariates had moderate or strong marginal associations with oxidative stress (*p*-value < 0.1). (**B**) Box and Whisker Plot quantitating endothelial mRNA expression of Catalase, Superoxide Dismutase, Heme Oxygenase-1, Thioredoxin Reductase-1 and NAD(P)H Quinone Oxidoreductase-1 in endothelial cells harvested from participants after adequate sleep (black dots; n = 28, 28, 25, 18 and 18, respectively) and sleep restriction (red dots; n = 32, 32, 25, 16 and 15, respectively). All data throughout the figure are shown as means ± SD (linear mixed effect model). NS = not significant.

To assess directly endothelial responses to sleep restriction, we used a minimally invasive technique to harvest venous ECs that can be directly examined without the artifact of culture conditions and have similar dysfunctional responses as arterial endothelium in cardiovascular disease^19–24^. We first assessed whether sleep restriction increases oxidative stress in ECs, as occurs in model organisms^11,17^. We used the redox sensitive fluorogenic probe CellRox that binds to nuclear DNA after being activated by reactive oxygen species^25^. Levels of oxidative stress were similar in ECs at baseline in adequate sleep and sleep restriction phase (fluorescence intensity [mean ± SD] 21.22 ± 16.43 vs. 24.22 ± 14.25, p = 0.49). The level of endothelial oxidative stress increased by 78% after sleep restriction compared with adequate sleep (fluorescence intensity [mean ± SD] 36.06 ± 21.55 vs. 20.23 ± 15.04, p = 0.002) and remained significantly greater after adjustment for baseline values (estimate ± SE 14.81 ± 4.41, p = 0.001) (Fig. 1A), indicating that mild, prolonged sleep restriction promotes endothelial oxidative stress in healthy female participants.

### Antioxidant responses are impaired in sleep restriction

Based on reports of increased antioxidant responses after sleep restriction in model organisms^17,26,27^, we anticipated upregulation of antioxidant genes in response to sleep restriction-induced endothelial oxidative stress in healthy female participants. As expected, exposure to oxidative stress by addition of hydrogen peroxide in cultured endothelial cells markedly upregulated expression of antioxidant genes (Supplementary Fig. S2). However, mRNA expression levels of ARE-containing genes Heme Oxygenase 1 (*HO-1)*, Thioredoxin Reductase 1 (*TXNRD-1*) and NAD(P)H Quinone Oxidoreductase 1 (*NQO-1*) as well as SuperOxide Dismutase 1 *(SOD1)* and *catalase*, markers of antioxidant enzymatic activity, were similar in ECs harvested from participants after adequate sleep and sleep restriction (Fig. 1B), suggesting that endothelial antioxidant responses are not appropriately activated in prolonged, mild sleep restriction.

Studies in Drosophila and rodent models have shown that sleep restriction upregulates antioxidant response via induction of the antioxidant regulator Nrf2, a redox sensitive transcription factor that translocates into the nucleus and initiates the transcription of antioxidant genes^12–15^. To probe potential mechanisms that might account for lack of antioxidant responses, we first investigated whether expression and cellular localization of Nrf2 are altered in ECs after sleep restriction. We confirmed that exposure to oxidative stress leads to increased nuclear localization of Nrf2 in cultured ECs (Supplementary Fig. S3). In contrast, in ECs harvested from participants, expression of Nrf2 mRNA and total protein was similar in adequate sleep and sleep restriction (Supplementary Fig. 4A–C). Whereas readily detected in cytoplasm, nuclear fluorescence of Nrf2 was almost undetectable in harvested ECs after both adequate sleep and sleep restriction (Supplementary Fig. S4D), indicating that Nrf2 does not translocate into the nucleus of ECs after sleep restriction despite increased endothelial oxidative stress.

### Endothelial Nrf2 cellular localization in sleep restriction

**Figure 2:**
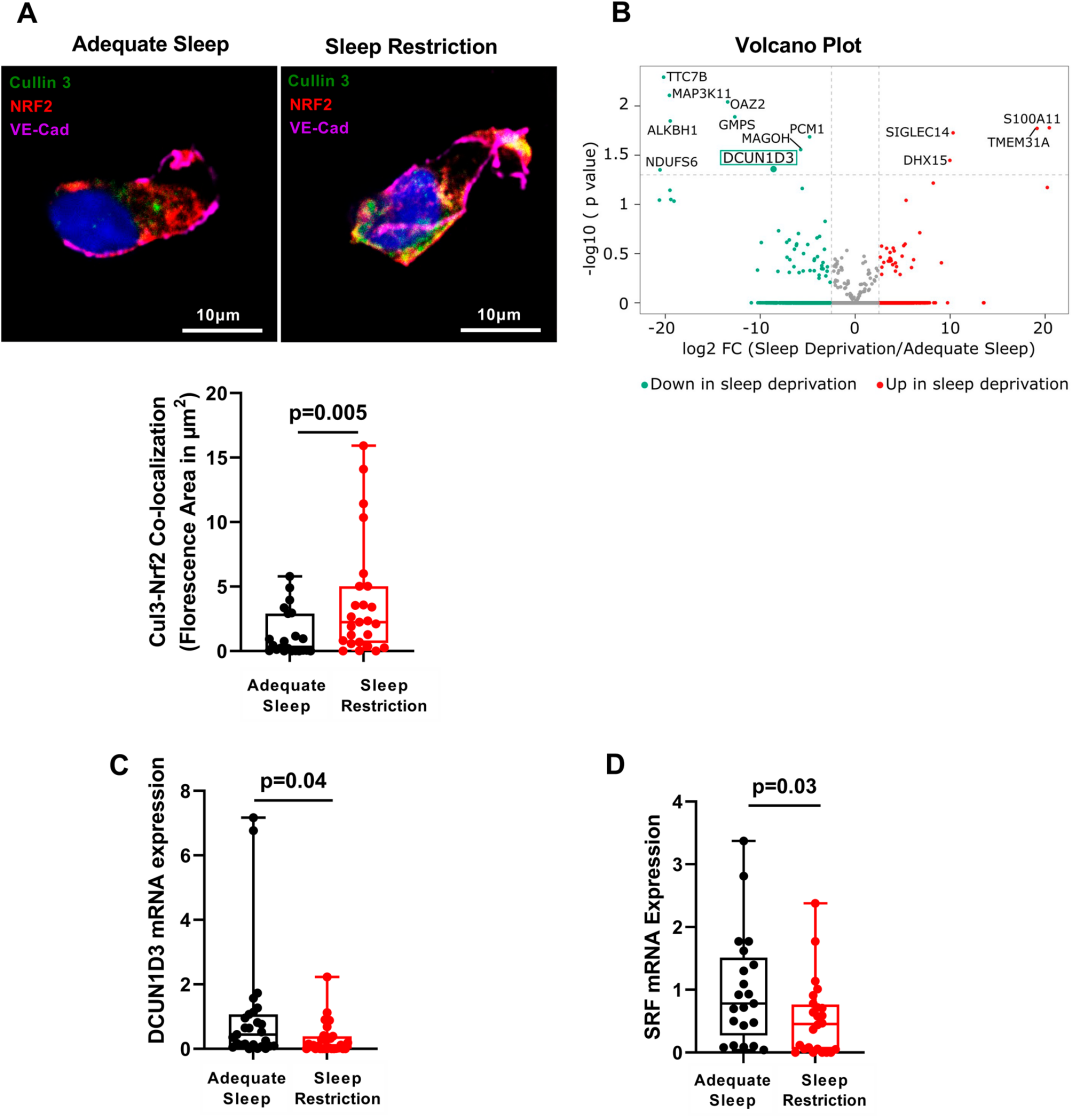
Identification of mediators of impaired antioxidant response in sleep restriction. (**A**) Representative images and Box and Whisker Plot quantitating endothelial Nrf2-Cul3 co-localization (indicated in yellow) after adequate sleep and sleep restriction (n = 25). (**B**) Volcano plot showing differential gene expression (n = 25,001 genes) in endothelial cells harvested from healthy participants (n = 5) after sleep restriction compared with adequate sleep. Horizontal dotted line separates 13 differentially expressed genes with *p* < 0.05 from those with *p* ≥ 0.05. Expression of *DCUN1D3* gene (green box), the only binding target of Cul3 among genes altered by sleep restriction, was reduced − 8.46 log2 fold change (FC) after sleep restriction compared with adequate sleep (*p* = 0.045). (**C**) Box and Whisker Plot quantitating endothelial *DCUN1D3* mRNA expression after adequate sleep (n = 28) and sleep restriction (n = 32). (**D**) Box and Whisker Plot quantitating endothelial *SRF* mRNA expression after adequate sleep and sleep restriction (n = 25). All data throughout the figure are shown as means ± SD (linear mixed effect model). Cul3 = Cullin-3; DCUN1D3 = defective in cullin neddylation 1 domain containing 3; Nrf2 = Nuclear factor (erythroid-derived 2)-like 2; SRF = serum response factor.

We next investigated why Nrf2 fails to transfer into the nucleus of harvested ECs after sleep restriction. Nrf2 function is regulated by the Cul3-Keap1-E3 ligase, a part of the Cullin-Ring-Ligase complexes^14^. Under basal conditions, Cul3 neddylation activates the E3-Cul3-Keap1 complex, which ubiquitinates Nrf2 and targets it for proteasomal degradation thereby maintaining the low basal levels of Nrf2^14,28,29^. Under conditions of increased oxidative stress, ubiquitination of Nrf2 is suppressed, resulting in increased availability of Nrf2 and its translocation into the nucleus, binding to ARE and consequent activation of the antioxidant genes^14,28,29^. Both protein and mRNA expression of Cul3 were similar in adequate sleep and sleep restriction (Supplementary Fig. S5A–B). In contrast, cytoplasmic co-localization of Cul3 and Nrf2 was significantly increased after sleep restriction compared with adequate sleep **(**Fig. 2A**)**, suggesting increased Nrf2 retention within its ubiquitination complex.

### Mediators of antioxidant responses in sleep restriction

To identify potential mediators of the altered Nrf2 and Cul3-Keap1-E3 complex interaction, we performed bulk RNA-seq in ECs harvested at the end of adequate sleep phase and sleep restriction phase in 5 participants (Supplementary Table S1). Sleep restriction altered expression of 13 genes (Fig. 2B). Using the predicted protein–protein interaction database (BioGRID)^30^, we interrogated protein products of those 13 genes for binding probability specifically to Nrf2, Keap1 and Cul3. While none were predicted to bind to Nrf2 or Keap1, we identified Defective in Cullin Neddylation-1 Domain Containing 3 (DCUN1D3) as the binding partner of Cul3 among genes altered by sleep restriction. Cul3 was the top predicted binding partner of DCUN1D3^30^. In non-endothelial cells, DCUN1D3 sequesters Cul3 to the plasma membrane thereby preventing its neddylation, reducing Nrf2 degradation and facilitating Nrf2 nuclear translocation and activation of antioxidant response^31,32^. We confirmed that DCUN1D3 interacts with Cul3 in human umbilical vein endothelial cells (HUVECs) indicating a function similar to that described in other cell types (Supplementary Fig. S6A). In RNA-seq analysis of ECs harvested from participants, endothelial *DCUN1D3* mRNA expression was reduced after sleep restriction compared with adequate sleep (log2 fold change = − 8.46; *p* = 0.045), which was confirmed by RT-PCR (Fig. 2C). Reduced expression of endothelial *DCUN1D3* coupled with increased interaction between Cul3 and Nrf2 suggest reduced sequestration of Cul3 to the plasma membrane and its greater availability in the Cul3-Keap1-E3 ubiquitin ligase complex, which traps Nrf2 thereby precluding Nrf2 nuclear translocation and activation of endothelial antioxidant response in sleep restriction.

### Regulation of DCUN1D3 in sleep restriction

We next investigated potential mechanisms linking sleep restriction to reduced expression of *DCUN1D3*. *DCUN1D3* gene was first identified during high-throughput screening of novel human genes that contain serum response element (SRE)^33,34^. SRE binds serum response factor (SRF), a transcription factor that regulates a variety of cellular processes^35^. Interestingly, SRF has recently emerged as a leading candidate transcription factor for priming the cerebral cortex response to short-term sleep restriction in mice^36^. SRF plays a key role in activity-dependent modulation of synaptic strength and its ortholog *blistered* is required to increase sleep duration after social enrichment in Drosophila^35,37,38^. Expression of SRF follows circadian pattern and its abundance is reduced during the wake period following a short-term sleep restriction in mice, which corresponds with the timing of EC harvesting in our participants^36^. Indeed, expression of *SRF* mRNA in harvested ECs was reduced after sleep restriction compared with adequate sleep (Fig. 2D), suggesting that curtailing sleep by delaying bedtime alters expression of a transcription factor that regulates DCUN1D3. Interestingly, binding of SRF to SRE, in coordination with other transcription factors, is required for activation of growth hormone (GH)-responsive genes that contain SRE^39^. Since DCUN1D3 contains SRE, we investigated whether its regulation by SRF is mediated by GH. This is of interest because a major pulsatile release of GH occurs during the slow wave sleep immediately after the sleep onset and sleep restriction blunts GH release^40^. The number of GH pulses and GH release are greatest for both sexes between 2300 and 0200 h^41^. Therefore, delaying bedtime by 1.5 h for 6 weeks may have disturbed pulsatile release of the GH in our participants. Because GH assessment requires frequent blood sampling over 24 h, which is not feasible in a prolonged, outpatient study, we incubated HUVECs with GH to assess its effects on *SRF* and *DCUN1D3* expression. As expected, addition of GH did not alter mRNA expression of *SRF*; however, it upregulated mRNA expression of *DCUN1D3* (Supplementary Fig. 6B). Silencing of *SRF* in HUVECs using siRNA (Supplementary Fig. 6C) suppressed mRNA expression of *DCUN1D3* both at baseline and after a 4 h exposure to oxidative stress compared with controls (Supplementary Fig. S6D), suggesting that SRF indeed regulates its expression. Interestingly, mRNA expression of *DCUN1D3* both at baseline and after a 4 h exposure to oxidative stress remained suppressed even after addition of GH in HUVECs with *SRF* knockdown compared with control (Supplementary Fig. S6D), indicating that SRF mediates effects of GH on DCUN1D3 expression.

**Figure 3:**
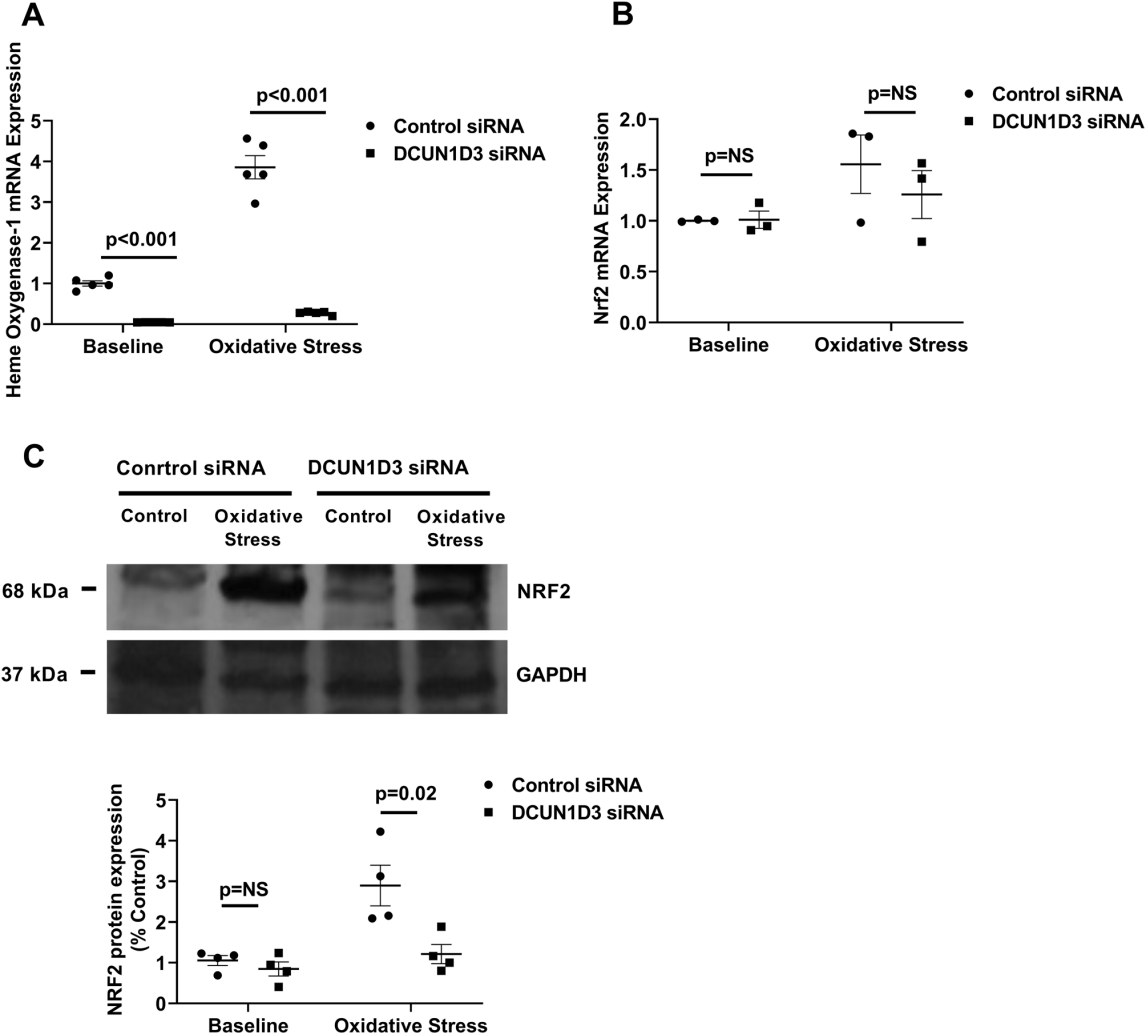
DCUN1D3 Regulates endothelial antioxidant response. (**A**) Scatter plot quantifying Heme Oxygenase-1mRNA expression before and after exposure to oxidative stress in HUVECs with *DCUN1D3* knockdown and control (n = 5). (**B**) Scatter plot quantifying *Nrf2* mRNA expression at baseline and after exposure to oxidative stress in HUVECs with *DCUN1D3* knockdown and control (n = 3). (**C**) Western Blotting and Scatter Plot quantifying Nrf2 protein expression in *DCUN1D3* knockdown compared to control at baseline and after exposure to oxidative stress in HUVECs (n = 4). All data throughout the figure are shown as means ± SD. NS = non-significant. Abbreviations as in Fig. 2.

To investigate whether DCUN1D3 independently regulates antioxidant response in ECs, we silenced *DCUN1D3* in HUVECs using siRNA (Supplementary Fig. S7). Remarkably, the expression of *HO-1* mRNA, a major ARE-containing Nrf2 target gene, in response to oxidative stress was almost completely abrogated in HUVECs with *DCUN1D3* knockdown (Fig. 3A), suggesting that DCUN1D3 independently regulates Nrf2-mediated antioxidant response in ECs. These finding suggest that DCUN1D3 is required for activation of Nrf2-mediated antioxidant response in ECs. In addition, *NRF2* mRNA expression was similar in HUVECs with silenced *DCUN1D3* and controls both at baseline and after exposure to oxidative stress (Fig. 3B). NRF2 protein expression was similar in HUVECs with *DCUN1D3* knockdown and controls at baseline. However, after exposure to oxidative stress, NRF2 protein expression was significantly reduced in HUVECs with *DCUN1D3* knockdown compared with controls (Fig. 3C), suggesting that NRF2 may indeed undergo enhanced proteasomal degradation when endothelial expression of *DCUN1D3* is reduced as seen in sleep restriction.

## Discussion

**Figure 4:**
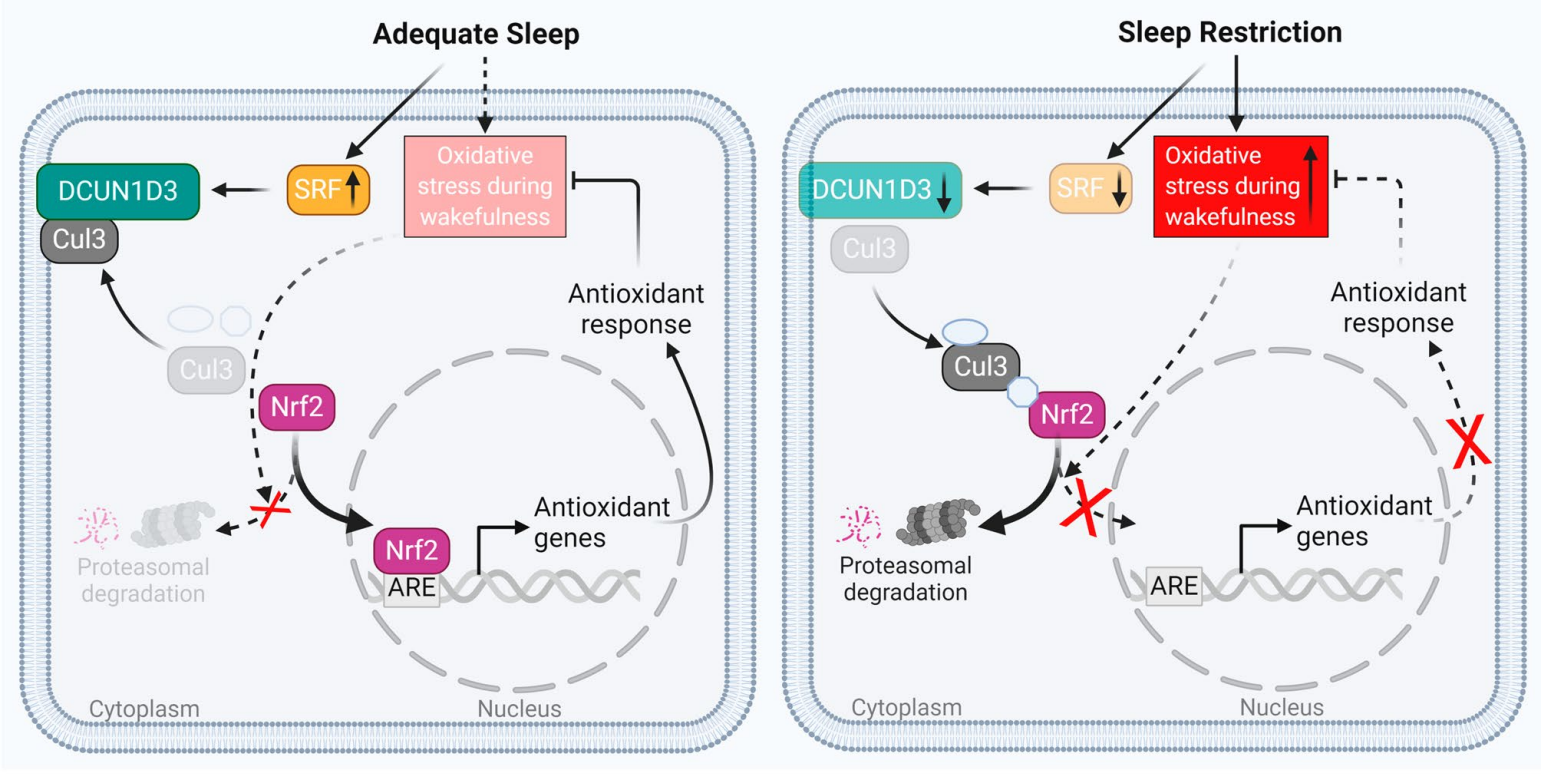
Endothelial cell function during wakefulness is impaired after sleep restriction compared with adequate sleep. After adequate sleep, endothelial oxidative stress that accumulates during wakefulness is cleared by an appropriate antioxidant response. *SRF* mRNA expression increases after sleep pressure build-up during wakefulness, which upregulates DCUN1D3 and sequesters Cul3 toward plasma membrane. Reduced Cul3 availability in the Nrf2 ubiquitination complex releases Nrf2 and allows for its nuclear translocation and activation of antioxidant genes. After sleep restriction, endothelial antioxidant responses are not appropriately upregulated leading to increased endothelial oxidative stress. Reduced *SRF* mRNA expression during wakefulness after sleep restriction leads to reduction in *DCUN1D3* expression and consequent increase in Cul3 availability in Nrf2 ubiquitination complex, which traps Nrf2 and precludes its nuclear translocation and activation of antioxidant genes. Impaired endothelial antioxidant response after insufficient sleep results in increased oxidative stress during wakefulness, which impairs endothelial function and may increase cardiovascular risk.

We used a rigorous, randomized crossover design and freshly harvested ECs to show directly that insufficient sleep increases endothelial oxidative stress in healthy female persons. Remarkably, endothelial antioxidant responses were completely lacking after sleep restriction despite markedly increased endothelial oxidative stress. We identified reduced expression of endothelial DCUN1D3, a protein that facilitates Nrf2-mediated antioxidant response in ECs, as a novel mechanism mediating the lack of endothelial antioxidant response to sleep restriction-induced oxidative stress. Curtailing sleep by delaying bedtime reduced expression of endothelial DCUN1D3 regulator SRF, a transcription factor that primes cortical response to sleep restriction^36^. These findings provide direct evidence that curtailing sleep, a highly prevalent behavioral pattern among adults, has detrimental effects on vascular health (Fig. 4).

Insufficient sleep has been long-linked to increased intracellular oxidative stress in model organisms. Short-term sleep restriction impairs the mitochondrial electron transport chain and increases reactive oxygen species production in Drosophila and mouse models thereby increasing oxidative stress^11,42,43^. A pro-inflammatory transcription factor NF-κB is in part regulated by the redox status of the cell and reactive oxygen species activates NF-κB in venous ECs^44^. We reported recently that mild, prolonged sleep restriction activates NF-κB in ECs, suggesting a mechanistic link between increased oxidative stress and inflammation in ECs after sleep restriction in healthy female persons^7^. Female persons have a greater pro-inflammatory response than males both during adequate and restricted sleep, suggesting that this may be an important underlying mechanism responsible for the sex difference in cardiovascular risk associated with insufficient sleep^4,5,45^.

We have identified DCUN1D3, a protein that sequesters Cul3 to the plasma membrane, as a novel mediator of impaired antioxidant response in insufficient sleep. Cul3-containing ubiquitin ligase complex targets Nrf2, a major activator of antioxidant response, for proteasomal degradation. *Insomniac* mutation causing short sleep in Drosophila leads to loss of ubiquitin ligase adaptor function and deletion of *Cul3* mimics short sleeping phenotype in Drosophila^46^. Reduced expression of DCUN1D3 in sleep restriction precludes Cul3 sequestration to the plasma membrane thereby allowing for continuous Nrf2 interaction with its Cul3-containing ubiquitin ligase complex, which prevents activation of antioxidant genes^32,47–50^. Our data link delaying bedtime with downregulation of SRF, a DCUN1D3 activator^31,32^. SRF is a transcription factor under the circadian control of sleep pressure and insufficient sleep reduces SRF expression during wakefulness^36^. Curtailing sleep by delaying bedtime alters release of GH, which may have downstream suppressive effects on SRF/DCUN1D3/Cul3/Nrf2-mediated antioxidant response in ECs^40^. Thus, we have identified novel axis linking insufficient sleep to SRF/DCUN1D3/Cul3/Nrf2-mediated antioxidant response in ECs.

The lack of endothelial Nrf2 nuclear translocation in sleep restriction raises a question of possible therapeutic application of Nrf2 activators^51,52^. Low dose of Nrf2 activator dh404 attenuates atherosclerosis in diabetic mice and its antioxidant and anti-inflammatory properties correlate with improvements in diabetes-associated atherosclerosis^53^. However, the safety profile of Nrf2 activators needs to be improved before contemplating their therapeutic role in insufficient sleep^51,52^. Our findings of endothelial dysfunction caused by insufficient sleep in apparently healthy female persons highlight the importance of counseling individuals at their health care encounters about the importance of adequate sleep for cardiovascular health.

Study limitations include the use of venous endothelial cells, which preclude conclusions regarding atherosclerosis in sleep restriction. However, inflammatory and oxidative pathways are activated similarly in venous and arterial ECs in healthy subjects and patients with atherosclerosis and venous and arterial ECs are exposed to the same circulating environment in adequate sleep and sleep restriction^22–24^. This study does not address potential atherogenesis after sleep restriction. Endothelial biopsy at specific sites of the arterial vasculature that are susceptible to atherosclerosis would be required to determine the precise mechanisms underlying atherosclerosis in response to sleep restriction, which is not feasible in an outpatient study involving healthy individuals. The lack of male persons precludes conclusions regarding sex differences in endothelial responses to insufficient sleep. We did not monitor participants’ menstrual cycle throughout this study; however, we enrolled premenopausal participants with normal duration of the menstrual cycle to maintain consistent hormonal status at study endpoints. The endpoints of each sleep phase were separated by 12 weeks, corresponding with the duration of 3 normal menstrual cycles.

In conclusion, insufficient sleep promotes endothelial oxidative stress and impairs antioxidant responses, suggesting that curtailing sleep leads to endothelial dysfunction that over time increases cardiovascular risk.

## Methods

Detailed methods are in the Supplementary Information.

All methods were performed in accordance with the relevant guidelines and regulations.

Participants were enrolled into the clinical trial NCT02835261 starting 01/09/2016.

### Study participants

Study participants were prospectively recruited from the community through advertising. Healthy female participants (i.e., persons whose female sex was assigned at birth) aged > 18 years with adequate sleep duration defined as 7–9 h/night were eligible for the study. Adequate sleep duration was confirmed by a 2 week actigraphy monitoring during screening. Exclusion criteria were history of any medical, neurological or psychiatric condition, eating or sleep disorder, regular use of any medications or supplements (including oral contraceptives and hormone replacement therapy), pregnancy within 1 year or active nursing, irregular menstrual cycle (< 28 or > 35 days), BMI < 20 or > 33.0 kg/m^2^, history of smoking, alcohol or drug abuse, shift work, daytime napping, travel across time zones within 4 weeks prior to screening and employment as a machine operator or commercial driver. Additionally, participants were not eligible for the study if they had poor sleep quality (Pittsburgh Sleep Quality Index score > 6), excessive daytime sleepiness (Epworth Sleepiness Scale score > 10) or increased risk for sleep apnea (Berlin Questionnaire score > 1). This study was approved by the Columbia University Institutional Review Board and all participants gave written informed consent.

Fifty-one participants were enrolled between September 2016 and December 2019. Sixteen participants did not complete at least one study phase and had no data available for intention-to-treat analysis. Thirty-five participants completed at least one phase of the study. Three participants completed adequate sleep but not sleep restriction phase; 32 completed both phases of the study (Supplementary Fig. S1). Demographic characteristics and sleep duration during screening for 16 participants who dropped out and 35 who completed at least one study phase were similar as reported previously^7^.

### Sleep restriction Intervention

We used actigraphy to objectively assess sleep duration during this study. After completing a 2 week actigraphy screening to determine whether their sleep duration is adequate (7–9 h daily), participants were randomly allocated to a 6 week adequate sleep phase (sleep duration between the woman’s regular bed- and wake-times that were determined during a 2 week actigraphy screening) or a 6 week mild sleep restriction phase (delaying bedtime by 1.5 h and keeping wake-time constant) followed by a 6 week washout period and crossover to the alternate sleep phase. During the adequate sleep phase, participants were asked to follow a fixed bedtime routine based on their 2 week screening sleep schedule. During the sleep restriction phase, participants were asked to delay their bedtime by 1.5 h and keep their habitual wakeup time unchanged.

Participants were randomized to a sleep phase sequence, either adequate sleep-sleep restriction or sleep restriction-adequate sleep, using an online research randomizer (https://www.randomizer.org). Participants remained blind to their randomization sequence until the start of the study. Systolic and diastolic blood pressure, BMI, plasma cortisol levels and actigraphy-derived physical activity and energy expenditure (step count [step/day], moderate-vigorous physical activity [min/day] and metabolic equivalents [kcal/kg/h]) were not significantly affected by sleep restriction as reported previously^7^.

### Vascular endothelial cell harvesting

ECs collection was performed between 10:00 and 11:00 AM in a fasting state at the beginning and end of both study phases. A 20-gauge angiocatheter was inserted into a forearm vein. Under sterile conditions, 3 J-shaped vascular guide wires (Arrow, Reading, PA) were sequentially advanced into the vein up to 10 cm. Tips of the wires were removed and washed in EC dissociation buffer kept at 4 °C. Each harvesting yielded 2000–5000 ECs^19,21^.

### Immunofluorescence

Nuclear fluorescence intensity of the fluorogenic probe activated by reactive oxygen species and subsequent binding to DNA, a marker of oxidative stress was assessed using fluorescence microscopy. Nrf2 and Cul3 fluorescence area were accessed in harvested ECs by confocal microscopy. ImageJ was used for quantification^19,21^.

### Quantitative real-time (RT)-PCR

Expression of messenger RNA (mRNA) isolated from the harvested ECs was quantified using quantitative RT-PCR (TaqMan One-Step RT-PCR Master Mix and TaqMan Probes, Applied Biosystems) for *SOD1, catalase, HO-1, TXNRD-1* and *NQO-1*, *Nrf2, Cul3, DCUN1D3* and *SRF* genes. Expression of *HO-1, DCUN1D3* and *SRF* mRNA was also quantified in HUVECs before and after exposure to hydrogen peroxide 200 µM for 4 h^22^. Results were expressed as Ct values normalized to the housekeeping gene *β-actin*.

## Statistical analysis

Two sample *t*-tests (for continuous variables) and Chi-square tests (for categorical variables) were used to determine whether baseline demographic characteristics are comparable between participants who were assigned a different order of the sleep intervention (i.e., habitual sleep followed by restricted sleep vs. restricted sleep followed by habitual sleep). By randomization, these two groups (i.e., habitual sleep first vs restricted sleep first) are expected to be homogeneous; however, they could differ by chance. To ensure that these 2 groups did not differ by chance, we assessed whether their baseline characteristics were comparable using the two-sample t-test. The baseline characteristics of these 2 groups were comparable. Normality was visually assessed by QQnorm plot. Owing to the nature of the crossover design, we assessed whether the endpoints in the second sleep phase were confounded from the order effect from the participant’s random allocation in the first sleep phase. The sleep phase assignment sequence was not significant for any outcome, indicating that data from the second phase are not confounded with residual effect from the first sleep phase (Supplementary Table S2).

The endpoints from each participant’s two sleep phases were averaged, and a linear model was used to regress it against the phase assignment sequence (adequate sleep first or sleep restriction first) while controlled for the participants’ baseline values. The sleep phase assignment sequence was not significant, indicating that no order effect confounded endpoints in the second sleep phase. Thus, the analysis was performed with data from both sleep phases using linear mixed effect model to take within-patient correlation into account. This was exploratory analysis for the clinical trial NCT02835261. The power analysis was calculated for the primary outcome (flow-mediated dilation of the brachial artery) of this trial, which was reported previously^7^.

We used linear mixed effect model to assess the effect of sleep restriction on markers of endothelial function at the end of adequate sleep and sleep restriction phase in intention-to-treat analysis. The main covariate of interest was the sleep phase (i.e., adequate sleep or sleep restriction). Participants were included in the model as random effect to account for within-subject correlations. To further adjust for potential confounding effects, controlled covariates (age; race; ethnicity; baseline and endpoint levels of BMI, systolic and diastolic blood pressure, and cortisol; actigraphy-derived physical activity and energy expenditure: step count (step/day), moderate-vigorous physical activity [min/day] and metabolic equivalents [kcal/kg/h]; an indicator of circadian phase: midsleep time change between phases; baseline values of endothelial markers) with moderate or strong marginal associations with the outcome (*p*-value < 0.1) were considered. We used regression coefficient plot to compare and visualize the effect of sleep restriction on endpoints. Considering that endpoints had different magnitude and scale, the regression coefficients *β* were normalized by standard errors of the corresponding endpoint thereby rendering standardized coefficients *β* comparable across endpoints. For in vitro data, *t*-test was used for all comparisons with a *p*-value cut off of < 0.05.

### Supplementary Information

Supplementary Information.

## Data Availability

The data underlying this article will be provided upon request from the corresponding author.
