# Supplementary material for: Mild sleep restriction increases endothelial oxidative stress in female persons
Source: Sci Rep. 2023 Sep 16;13:15360. doi: 10.1038/s41598-023-42758-y (PMC10505226; doi:10.1038/s41598-023-42758-y)
Supplement: Supplementary file 1 — Supplementary Information. [file 41598_2023_42758_MOESM1_ESM.docx]

**Mild Sleep Restriction Increases Endothelial Oxidative Stress in Female Persons**

**Supplementary Information**

Riddhi Shah^1^, Vikash Kumar Shah^1†^, Memet Emin^1†^, Su Gao^1^, Rosemary V. Sampogna^2^, Brooke Aggarwal^3^, Audrey Chang^4^, Marie-Pierre St-Onge^5^, Vikas Malik^1,6^, Jianlong Wang^1,6^, Ying Wei^7^ & Sanja Jelic^1^*

^1^Division of Pulmonary, Allergy, and Critical Care Medicine, ^2^Division of Nephrology, ^3^Division of Cardiology, ^4^NewYork-Presbyterian Morgan Stanley Children's Hospital, ^5^Division of General Medicine, ^6^Columbia Center for Human Development and Columbia Stem Cell Initiative, ^7^Division of Biostatistics, Columbia University Vagelos College of Physicians and Surgeons, New York, NY.

^†^Equal contribution

***Corresponding Author**:

Sanja Jelic, M.D.

Email: [sj366@cumc.columbia.edu](mailto:sj366@cumc.columbia.edu)

**Supplementary Information**

Expanded Methods

Supplementary Figures 1-12

Supplementary Tables 1-2

Supplementary References (56-61)

**Materials and Methods**

**Actigraphy monitoring**

Sleep duration was assessed objectively using actigraph GT3X+ monitors (Actigraph LLC, Pensacola, FL, USA). Monitors were worn on the non-dominant wrist 24 h/d during both phases and only removed to shower. Mean sleep duration was derived from the ActiLife software supplemented with information on bedtimes and wake times derived from sleep diaries completed by participants.

**Assessment of baseline sleep habits**

Sleep duration and quality were assessed using the validated Pittsburgh Sleep Quality Index (PSQI)^56^. The PSQI measures seven domains including subjective sleep quality, sleep onset latency, sleep duration, habitual sleep efficiency, sleep disturbances, use of sleep medication, and daytime dysfunction over the last month to distinguish between poor and good sleepers. A global sum >6 indicates poor sleep^56^. According to the Sleep Research Society/American Academy of Sleep Medicine consensus statement, adequate sleep should be ≥7 h/night, while sleeping <7 h/night is considered insufficient^57^. Participants were excluded from the study if they had poor sleep quality (PSQI score >6) or excessive daytime sleepiness (Epworth Sleepiness Scale score >10)^58^.

**Assessment of covariates**

A standardized health questionnaire was used to evaluate sociodemographic factors including age, race/ethnicity, as well as medical history. Height, weight and resting systolic and diastolic blood pressure were obtained by trained personnel. For blood pressure measurement, participants were sitting, legs uncrossed, with arms not restricted by clothing and relaxed, for at least 5 min before measurements were taken. The participant’s arm was supported at heart level, with palm up. Trained personnel palpated the brachial artery, positioned the cuff 1 inch above the site of brachial pulsation and centered the bladder of the cuff above the artery. The research grade automated blood pressure monitor was activated. A minimum of 2 readings were taken at intervals of at least 1 minute, and the average of those readings was used to represent the participant’s blood pressure. If there was a >5 mmHg difference between the first and second reading, an additional reading was obtained, and the average of the 3 readings was used^21^. Plasma levels of cortisol were measured in the Columbia University Biomarkers Core Laboratory.

**Vascular endothelial cell harvesting and isolation**

A 20-gauge angiocatheter was inserted into a superficial forearm vein. Under sterile conditions, 3 J-shaped vascular guide wires (Arrow, Reading, PA) were sequentially advanced into the vein up to 10 cm. ECs were retrieved from wire tips by washing with EC dissociation buffer. Harvesting yielded ~2000-5000 ECs^19,59,60^. For immunofluorescence, ECs were recovered by centrifugation at 4°C, 150G for 6 min, the cell pellet was re-suspended in red blood cell lysis buffer and incubated at 4°C for 5 min, then centrifuged at 150G for 6 min, fixed with 4% paraformaldehyde (Santa Cruz, Dallas, TX) in PBS for 10 min, washed twice with PBS, transferred to poly-L-lysine coated slides (Sigma, St. Louis, MO), and air dried at 37°C. The slides were stored at -80°C until analyzed. For mRNA extraction, the cell pellet was re-suspended with isolation buffer, incubated with biotinylated mouse anti-human monoclonal antibody directed against CD146 (Millipore, Temecula, CA, Cat. # 04-1147; 1:200) at 4°C for 15 min, then incubated at 4°C with Streptavidin FlowComp Dynabeads (Invitrogen, Solo, Norway; 1:100) for 45 min, followed by EC isolation by magnet^19^.

**Immunofluorescence**

Harvested ECs were identified by positive staining with goat anti-human polyclonal antibodies directed against CD144 (VE-Cadherin) (R&D Systems, Minneapolis, MN; Cat. # AF938; 1:20) followed by Texas Red-conjugated donkey anti-goat secondary antibodies (Jackson ImmunoResearch, 1:50) and stained with 4′,6-diamidino-2-phenylindole (DAPI) (Molecular Probes by Life Technology [now ThermoFisher Scientific, Waltham, MA] Cat. # D21490). At least 25 consecutive ECs were analyzed from each slide. Missing data are owing to the inability to identify at least 25 intact ECs per slide. For each protein, the number of samples with at least 25 intact ECs per slide available for analysis is reported in the corresponding figure legend. For oxidative stress, Cell ROX Green reagent (5µM, Invitrogen by ThermoFisher Scientific, Waltham, MA; Cat. # C10444), a fluorogenic probe activated by reactive oxygen species and subsequent binding to DNA, was used. Prior to assessing harvested ECs, we validated Cell ROX probe as a measurement of oxidative stress in HUVECs at baseline and after 4 h of exposure to 200 µM of H_2_O_2_. Compared with baseline, increased nuclear fluorescence intensity that was predominantly located in the nucleus was detected after 4 h exposure to 200 µM of H_2_O_2_ in HUVECs (**Supplementary Fig. S8**). Oxidative stress was quantified using florescence microscopy (Nikon Eclipse E600) and ImageJ. Nrf2 (Abcam, Cat. # ab89443) and Cul3 (Abcam, Cat. # ab194584) fluorescence area were accessed in harvested ECs by confocal microscopy. ImageJ was used for quantification^19,21^.

**RNA-seq analysis**

RNA libraries were prepared from 5 participants from ECs collected at the end of adequate sleep and the end of sleep restriction intervention. Each participant had 2 endothelial samples – at the end of adequate sleep and end of sleep restriction (total samples n=10). The quantity of total RNA used for bulk RNA-seq library preparation ranged from 500 pg to 2 ng. Total RNA was extracted using RNAqueous Micro Kit (Invitrogen by Thermo Fisher Scientific) according to manufacturer’s protocol. This kit is designed specifically for extraction of total RNA from micro-scale samples. Total RNA was converted to cDNA using the Ovation Pico WTA System V2 per manufacturer’s instructions. The cDNA was quantified using Biolanalyzer - High Sensitivity DNA Assay using Agilent technology at the Molecular Pathology Core Facilities at Columbia University Irving Medical Center. Next, cDNA was further sheared to 150 bp using DNA Shearing Covaris e220 machine for 6 min. Quantity of sheared DNA ranged from 5-16 ng and was used to prepare sequencing libraries using the Ultra II DNA Library Preparation Kit (NEBNext). NEBNext Adaptors were ligated to the DNA using ligation master mix compatible for Illumina sequencing followed by size selection of adaptor ligated DNA using magnetic beads. NEBNext multiplex Oligo’s were added to the adaptor ligated DNA by PCR amplification using NEBNext Index primers. Ten nM total sequencing library was prepared from 10 samples and further diluted to 1.6 pM concentration as required by the 2*75bp NextSeq 500 Mid Output Illumina Kit manual. We sequenced RNA-seq samples (76 bp, paired-end) with Illumina NEXTSEQ 500 platform, and analyzed using Illumina’s BaseSpace online tool Illumina TopHat Alignment v1.0.0 ([www.illumina.com](http://www.illumina.com)). The raw and processed files have been deposited to GEO (accession number GSE171831). Reads were aligned using TopHat Alignment v1.0.0 (reference genome: Homo sapiens/HG19(REFSEQ) and Trim Truseq Adapters inputs), and differential expression was calculated using Cufflinks Assembly & Differential Expression 1.1.0 (www.illumina.com). Cufflinks Assembly & Differential Expression app, a part of TopHat software, has integrated cuffdiff for differential gene expression (log2-fold change [FC]). For log2-FC with p-values greater than 0.999, the p-values in cuffdiff output files round-up to 1. The p-values of 1 correspond to 0 on –log10 scale. Therefore, the genes with log2-FC p-values greater than 0.999 fall on the zero value on the y-axis in the Volcano plot (**Fig. 2B**). Thirteen genes had log2-FC with a p<0.05. Quality control (QC) details for read mapping and base quality are available in **Supplementary Table S3**.

**Identification of binding partners for Cul3**

Predicted protein-protein database BioGRID (<http://www.thebiogrid.org>) was used to predict the highest probability binding partners for Cul3 from the differential expressed genes comparing adequate sleep and sleep restriction^33^. Harmonizome Database (<http://amp.pharm.mssm.edu/Harmonizome/>), a common platform for all databases interpreting genes and proteins collected of helped to confirm presence of SRF binding at SRE and regulating expression of DCUN1D3 by JASPAR Predicted Transcription Factor Targets database, http://amp.pharm.mssm.edu/Harmonizome/JASPAR+

Predicted+Transcription+Factor+Targets^33,61,62^.

**Quantitative Real-Time (RT)-PCR**

We used TaqMan (Life Technology) method on Applied Biosystems StepOne Plus Real-Time PCR System. Messenger RNA (mRNA) was extracted using RNAqueous Micro Kit (Invitrogen by Thermo Fisher Scientific) according to manufacturer’s protocol. The range of 10 pg – 2 μg of mRNA was used for first-strand cDNA synthesis with qScriptXTL cDNA SuperMix (Quantabio) according to the manufacturer’s protocol. The first strand cDNA was further diluted 2.5 times with Tris-EDTA buffer and stored in -80°C. For 20 μl real-time PCR reaction the following reagents were added: 10 μl TaqMan Gene Expression Master Mix (2X) (Life Technology), 1 μl TaqMan Gene Expression Assay (20X), 5 μl Nuclease-fee water and 4 μl cDNA. All the real-time PCR primers and probes span an exon junction to eliminate the contamination of genomic DNA. TaqMan primers and probes were used for *SOD1, catalase, HO-1, TXNRD-1, NQO-1, Nrf2, Cul3, DCUN1D3* and *SRF* genes. Missing data are owing to undetermined expression of the assessed genes. For each gene, the number of samples with detectable expression available for analysis is reported in the corresponding figure legend. Expression of *HO-1, Nrf2, DCUN1D3* and *SRF* mRNA was also quantified in HUVECs before and after exposure to hydrogen peroxide 200 µM for 4 hours^22^. Results were expressed by Ct values normalized to the housekeeping gene *β-actin*^19^*.*

**Cell culture, transfection and siRNA treatment**

HUVECs (PromoCell, # C12200) were cultured in EC medium with 2% endotoxin-free heat-inactivated fetal bovine serum (FBS; Gibco) until they reached the required 40-80% confluence for siRNA transfections. DharmaFECT transfection reagent (Formulation-4, Dharmacon Horizon) was used to deliver siRNA into cultured HUVECs. ON-TARGET plus Non-targeting pool siRNA (5nmol, Dharmacon, Cat. # D-001810-10-05) was used as negative control with ON-TARGET plus Human DCUN1D3 siRNA-SMARTpool (5nmol, Dharmacon, Cat. # L-018390-00-0005) and ON-TARGET plus Human SRF siRNA-SMARTpool (5nmol, Dharmacon, Cat. # L-009800-00-0005) siRNA's for *DCUN1D3* and *SRF* respectively. A final siRNA concentration of 25nM was added to each well. Successful transfection was confirmed by >70% reduction in DCUN1D3 and SRF protein expression in three independent experiments using Western blotting.

**Western blotting**

HUVECs were lysed on ice with cell lysis buffer (Cell Signaling Technology) supplemented with Pierce protease and phosphatase inhibitor (ThermoScientific). Protein amount in the lysate was determined by Pierce BCA protein assay (ThermoScientific) to ensure equal protein loading on gels. Protein samples were mixed with Laemmli Sample Buffer (BIO-RAD) and separated on PROTEAN TGX Precast Protein Gel (BIO-RAD). Standard immunoblotting procedure was performed based on manufacturer's instructions (BIO-RAD). The primary antibodies are as follows: DCUN1D3 (Santa Cruz, Cat. # SC-514506), Cul3 (Abcam, Cat. # ab194584), Nrf2 (Abcam, Cat. # ab89443), SRF (Cell Signaling, Cat. # 5147T) and loading control glyceraldehyde 3-phosphate dehydrogenase (GAPDH) (Abcam, Cat. # 131165). Band intensities were quantified using ImageJ software. All uncropped blots are presented in **Fig. S9-S12**.

**Supplementary Information References:**

56. Buysse, D.J. *et al.* The Pittsburgh Sleep Quality Index: a new instrument for psychiatric practice and research. *Psychiatry Res*. **28**, 193–213 (1989).

57. Watson, N.F. *et al.* Recommended Amount of Sleep for a Healthy Adult: A Joint Consensus Statement of the American Academy of Sleep Medicine and Sleep Research Society. *Sleep* **38**, 843-844 (2015).

58. Johns, M.W. A new method for measuring daytime sleepiness: the Epworth sleepiness scale. *Sleep* **14**, 540-545 (1991).

59. Jelic, S. et al. Inflammation, Oxidative Stress and Repair Capacity of the Vascular Endothelium in Obstructive Sleep Apnea. *Circulation* **117**, 2270-2278 (2008).

60. Jelic, S. *et al.* Vascular Inflammation in Obesity and Sleep Apnea. *Circulation* **121**, 1014-1021 (2010).

61. Zhou, Y. *et al.* Metascape provides a biologist-oriented resource for the analysis of systems-level datasets. *Nat. Commun.* **3**, 1523 (2019).

62. Mathelier, A. *et al.* JASPAR 2014: an extensively expanded and updated open-access database of transcription factor binding profiles. *Nucleic Acids Res.* **42**, D142–D147 (2014).

**
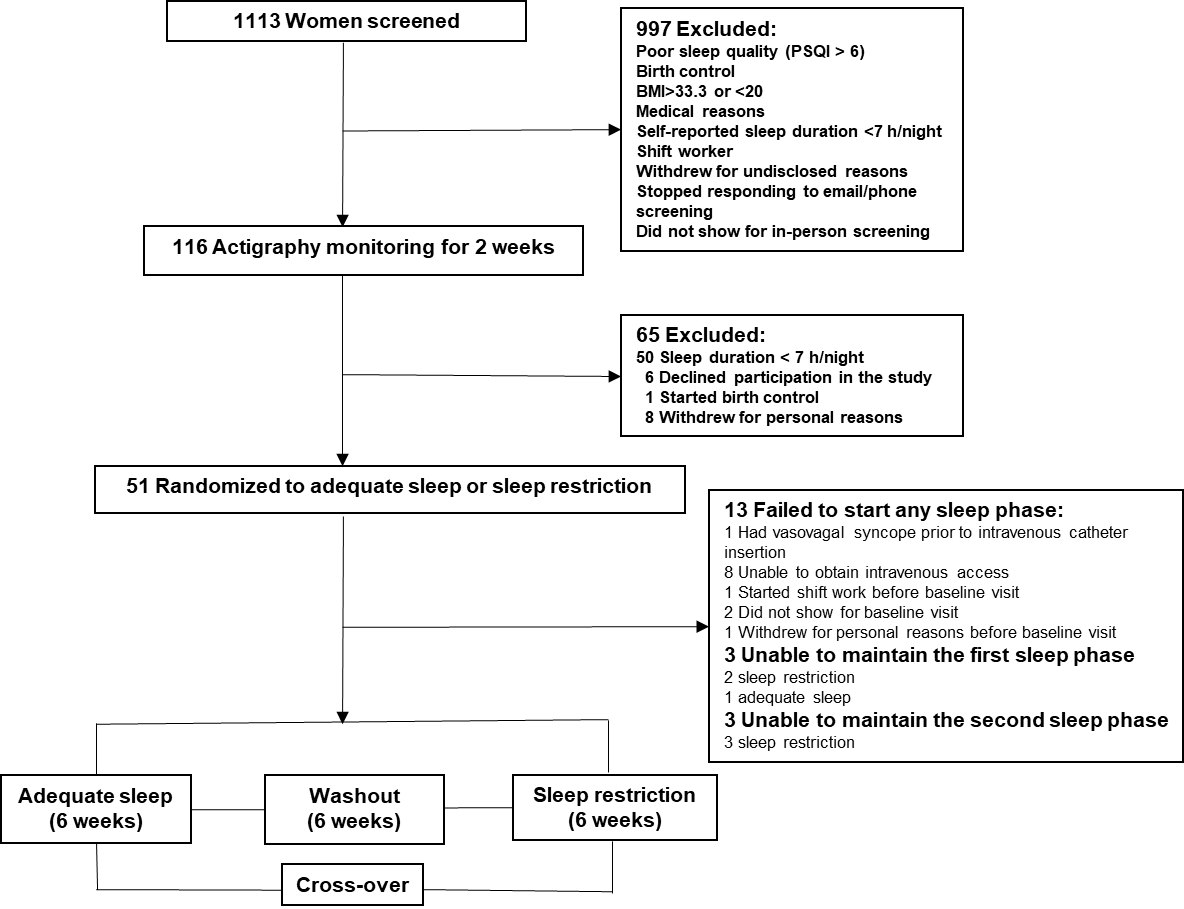
Supplementary Figures:**

**Supplementary Figure S1. Flow diagram and study protocol.**


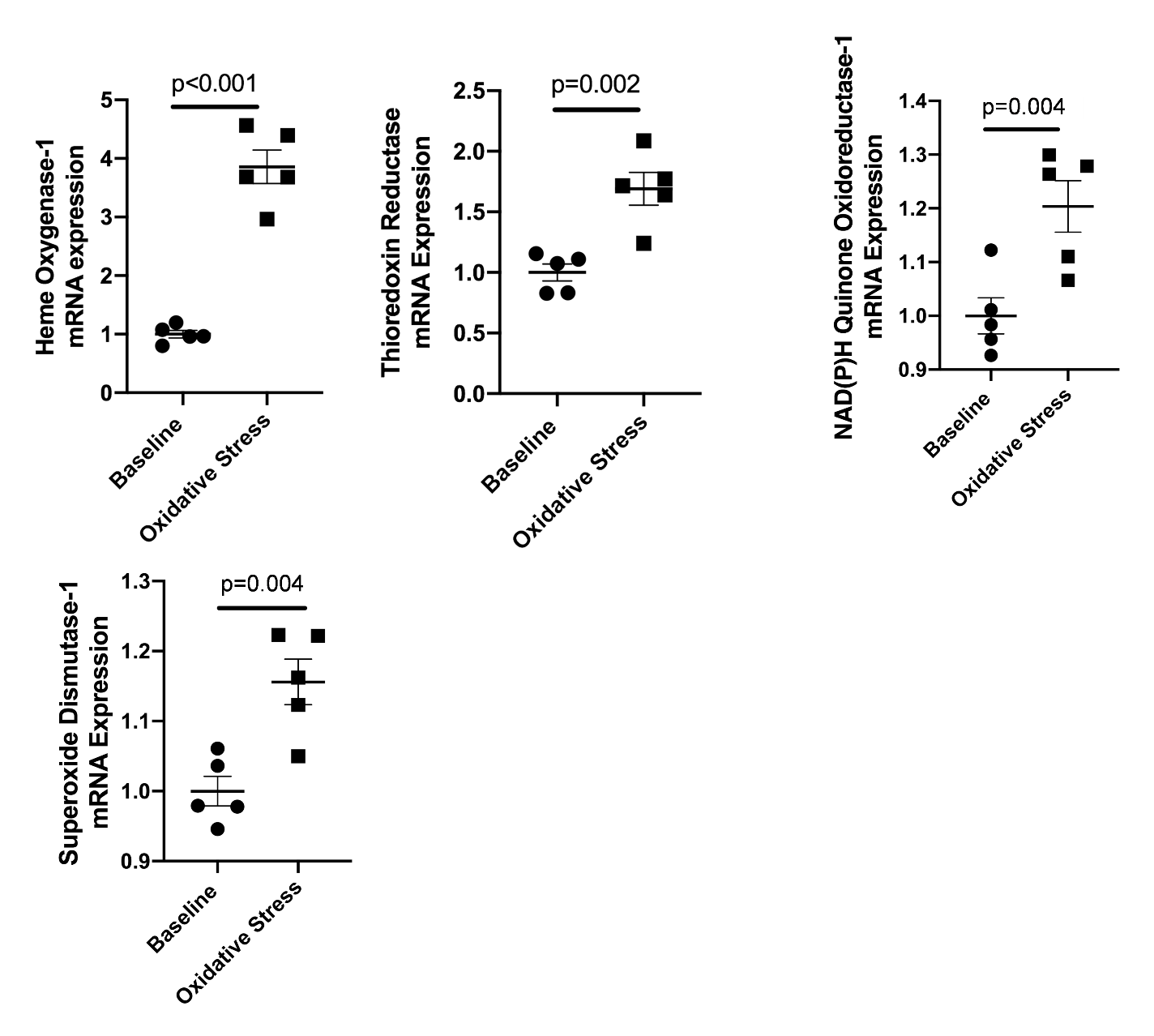


**Supplementary Figure S2. Exposure to oxidative stress increases antioxidant response in Human Umbilical Vascular Endothelial Cell (HUVECs).** Scatter Plot quantifying expression of antioxidant genes before (dots) and after (squares) exposure to oxidative stress (n=5). All data throughout the figure are shown as means ± SD.

**Supplementary** **Figure S3. Oxidative stress increases Nrf2 nuclear fluorescence in Human Umbilical Vascular Endothelial Cell (HUVECs).** Scatter Plot quantifying nuclear Nrf2 fluorescence before (dots) and after (squares) exposure to oxidative stress (n=3). All data throughout the figure are shown as means ± SD. Nrf2 = Nuclear factor (erythroid-derived 2)-like 2


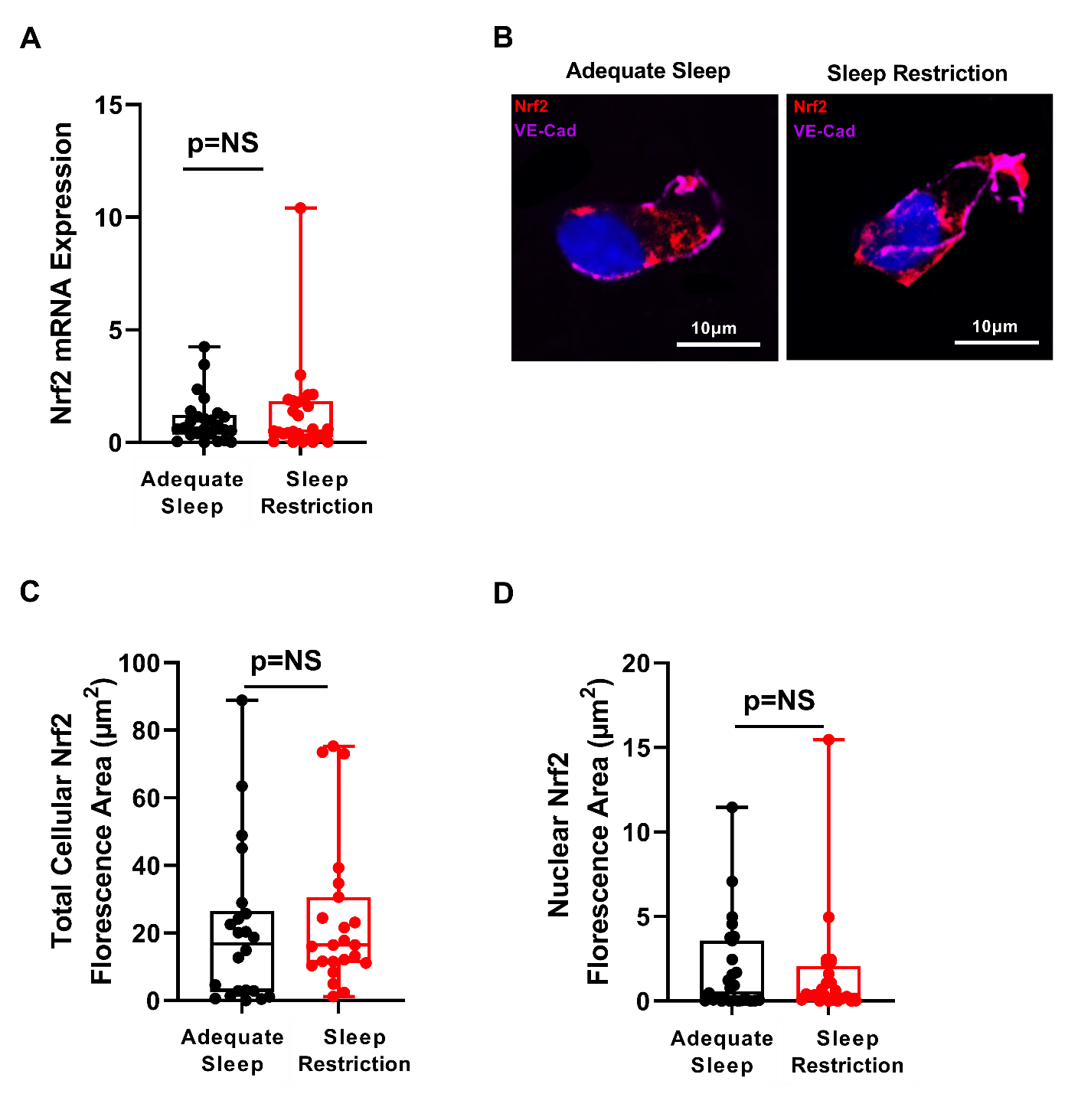


**Supplementary Figure S4. Nrf2 does not translocate into nucleus in response to sleep restriction-induced oxidative stress in endothelial cells. A)** Box and Whisker Plot quantitating endothelial mRNA expression of *NRF2* after adequate sleep (n=28) and sleep restriction (n=32). **B)** Representative images of endothelial total cellular and nuclear protein expression of Nrf2 after adequate sleep and sleep restriction. **C)** Box and Whisker Plot quantitating endothelial total cellular and **D)** nuclear protein expression of Nrf2 after adequate sleep and sleep restriction (n=25). All data throughout the figure (black dots = adequate sleep; red dots = sleep restriction) are shown as means ± SD (linear mixed effect model). NS = not significant. Nrf2 = Nuclear factor (erythroid-derived 2)-like 2


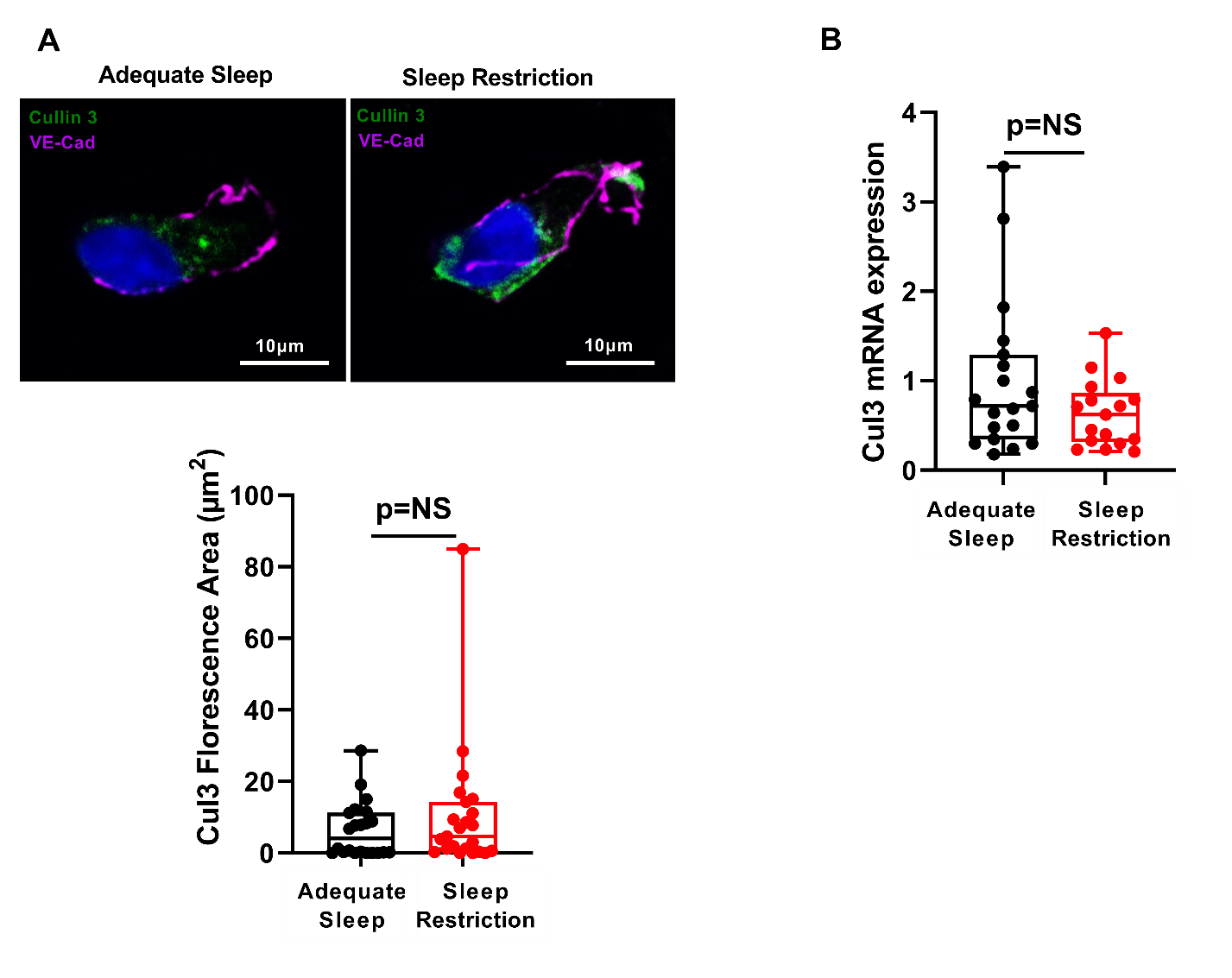


**Supplementary Figure S5. Sleep restriction does not alter Cul3 expression in endothelial cells. A)** Representative images and **B)** Box and Whisker Plot quantitating endothelial Cul3 protein and mRNA expression after adequate sleep (n=22 and 19, respectively) and sleep restriction (n=23 and 17, respectively). All data throughout the figure (black dots = adequate sleep; red dots = sleep restriction) are shown as means ± SD (linear mixed effect model). NS = not significant. Cul3 = Cullin-3


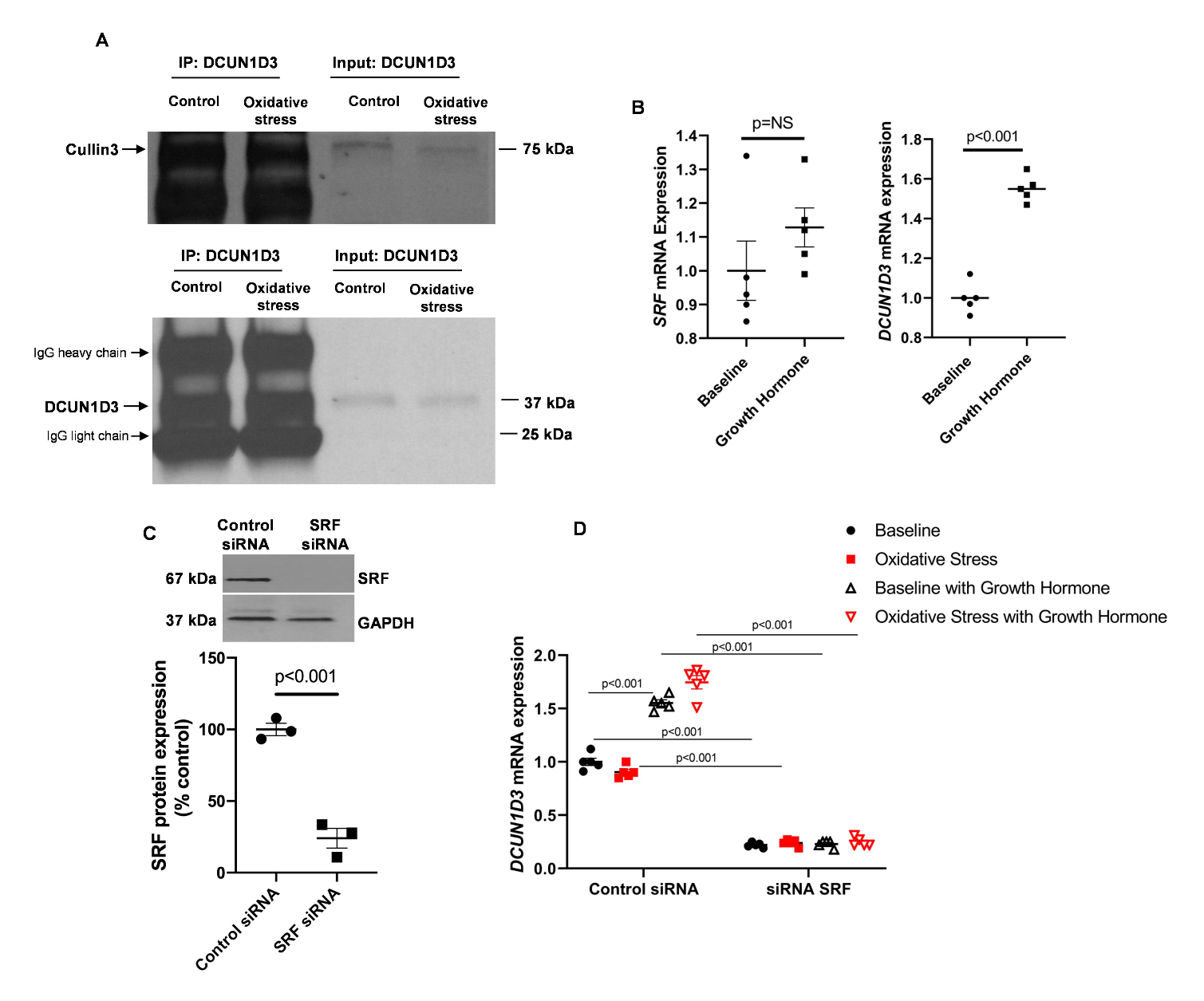


**Supplementary Figure S6. Growth hormone modulates SRF-DCUN1D3 interactions in Human Umbilical Vascular Endothelial Cell (HUVECs). A)** Co-immunoprecipitation of DCUN1D3 and Cul3 in HUVEC lysate followed by SDS-PAGE and Western blotting with anti-DCUN1D3 and anti-Cul3 antibodies, showing interaction between DCUN1D3 and Cul3. **B)** Scatter Plot quantifying expression of endothelial *SRF* and *DCUN1D3* genes after exposure to growth hormone (squares) compared to baseline (dots) (n=5). **C)** Western blotting and Scatter Plot quantifying SRF protein expression in *SRF* knockdown (squares) compared to control (dots) (n=3). **D)** *DCUN1D3* mRNA expression before and after exposure to oxidative stress and growth hormone in *SRF* knockdown HUVECs and HUVECs treated with control siRNA (n=5). All data throughout the figure are shown as means ± SD. NS = non-significant. Cul3 = Cullin-3; DCUN1D3 = defective in cullin neddylation 1 domain containing 3; SRF = serum response factor


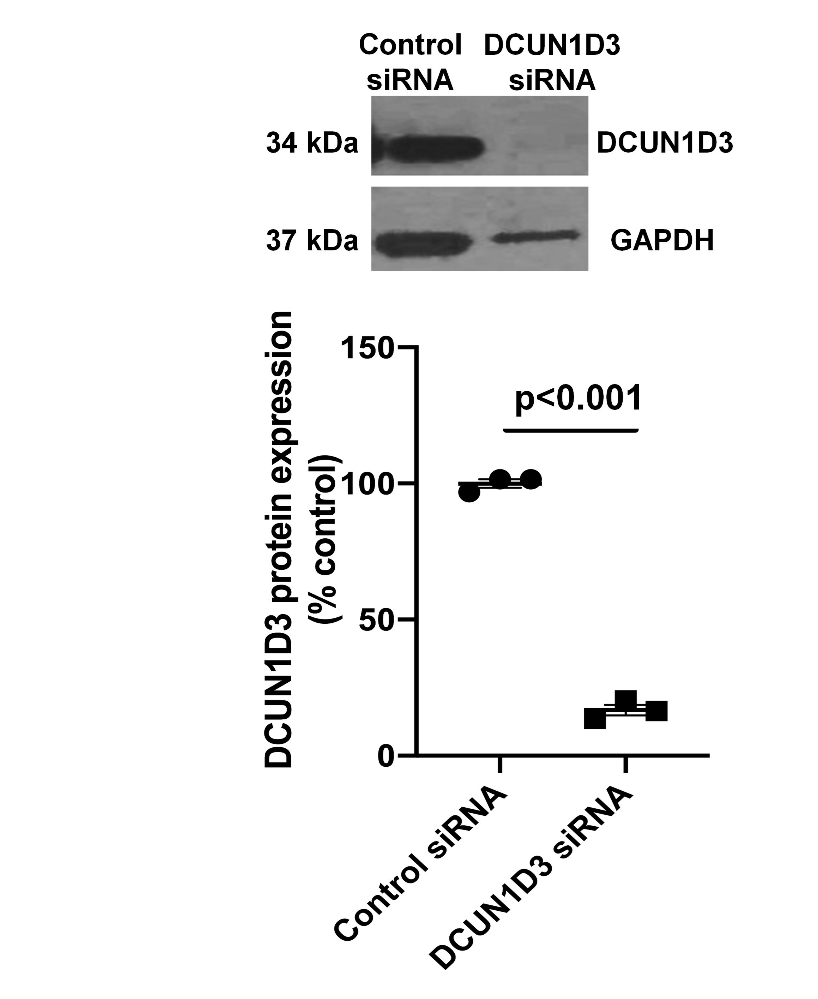


**Supplementary Figure S7. Silencing of *DCUN1D3* in Human Umbilical Vascular Endothelial Cell (HUVECs).** Western Blotting and Scatter Plot quantifying DCUN1D3 protein expression in *DCUN1D3* knockdown (squares) compared to control (dots) (n=3) in HUVECs. All data throughout the figure are shown as means ± SD. DCUN1D3 = defective in cullin neddylation 1 domain containing 3; GAPDH = glyceraldehyde-3-phosphate dehydrogenase


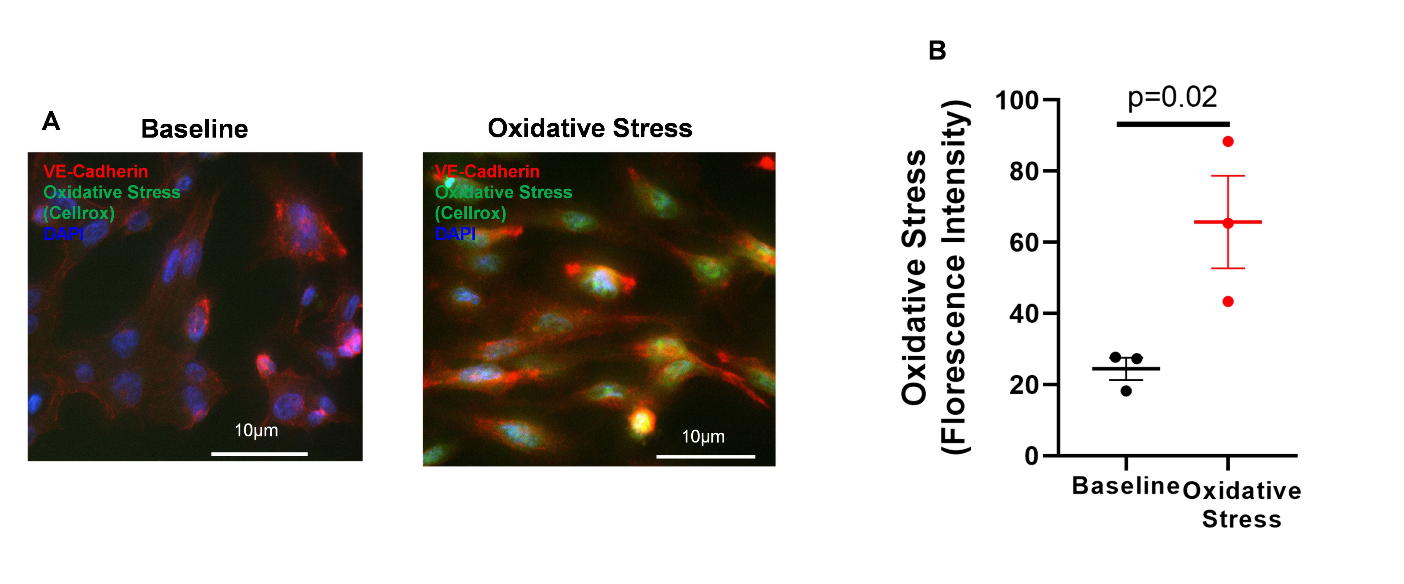


**Supplementary Figure S8. Validation of oxidative stress assessment in Human Umbilical Vascular Endothelial Cell (HUVECs). A)** Representative images and **B)** Scatter plot quantitating oxidative stress (nuclear fluorescence intensity of the fluorogenic probe activated by reactive oxygen species and subsequent binding to DNA) in HUVECs at baseline (black dots) and after exposure to hydrogen peroxide 200 µM for 4 h (red dots) (n=3). All data throughout the figure are shown as means ± SD.

**
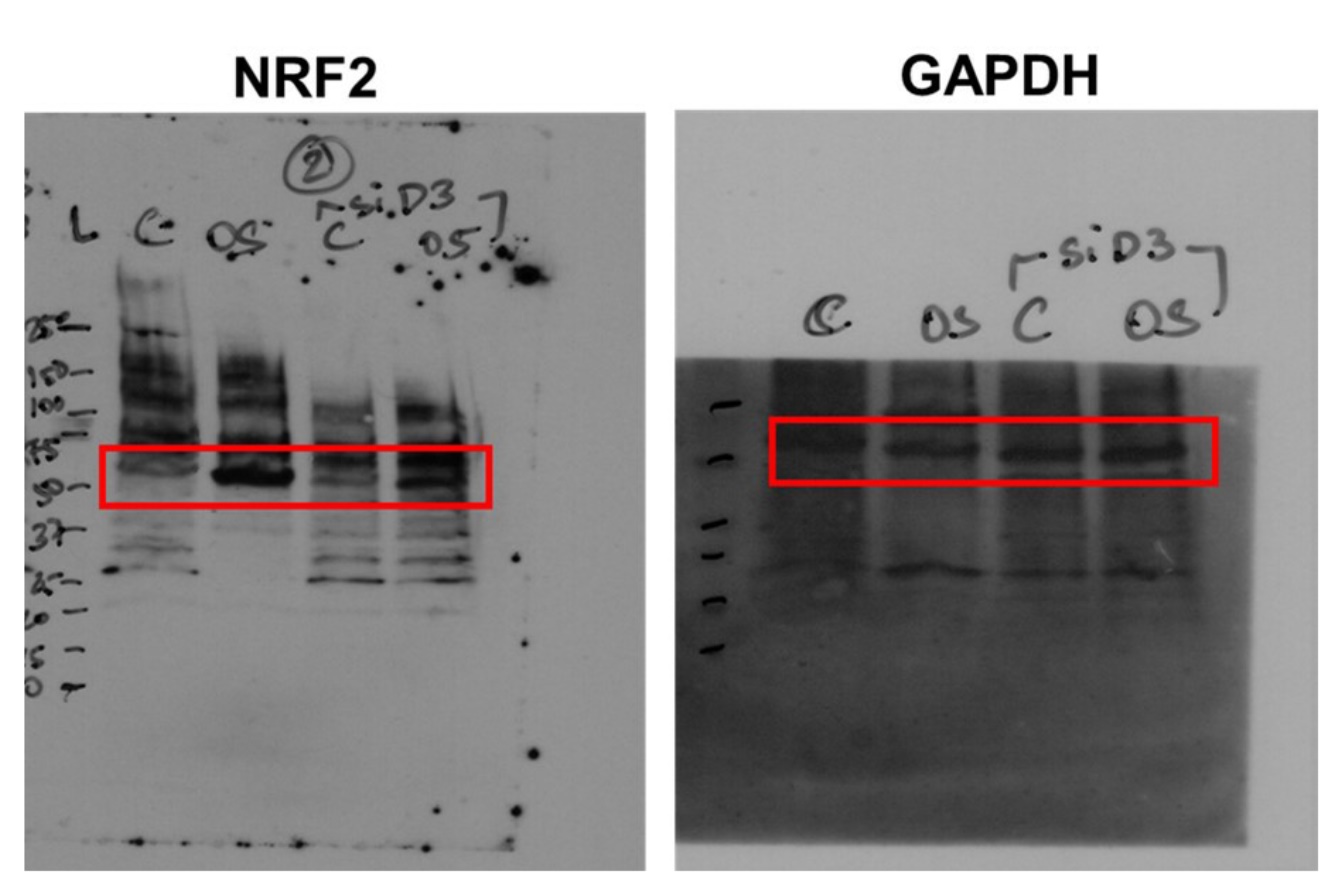
**

**Supplementary Figure S9.** Uncropped membrane stained with antibodies to NRF2 and GAPDH.

**
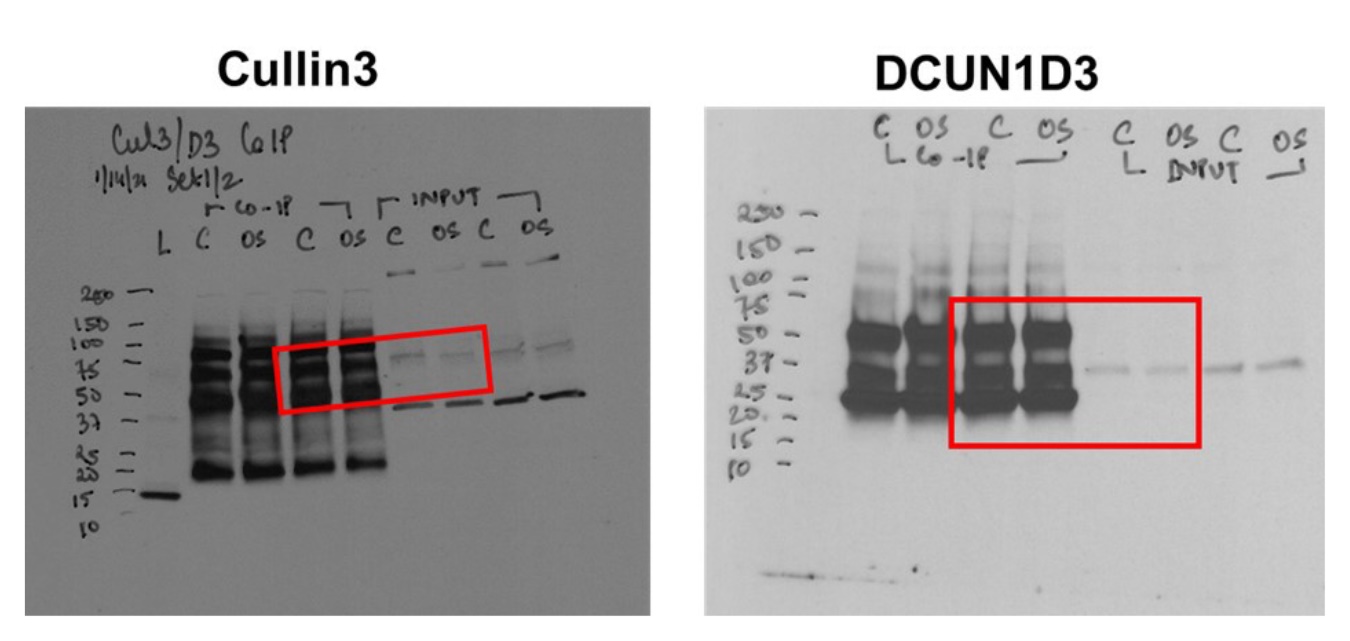
**

**Supplementary Figure S10.** Uncropped membrane of co-immunoprecipitation of DCUN1D3 and Cul3.

**
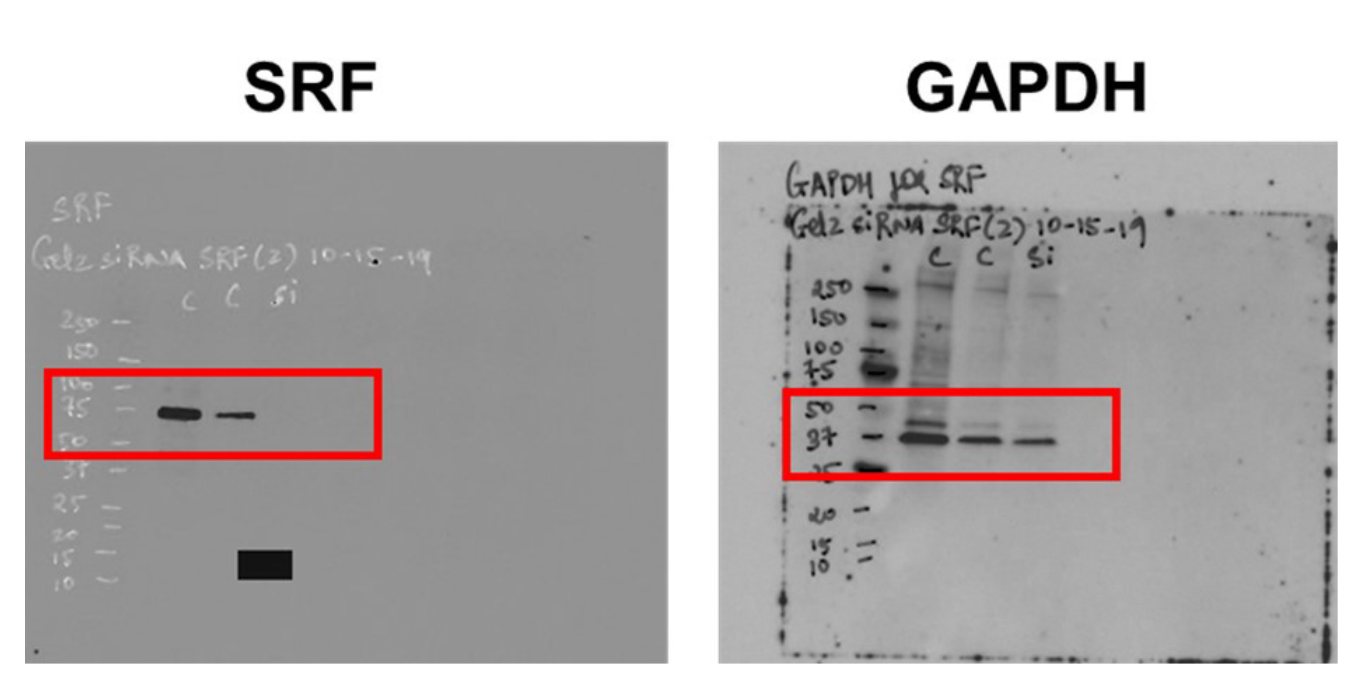
**

**Supplementary Figure S11.** Uncropped membrane stained with antibodies to SRF and GAPDH.

**
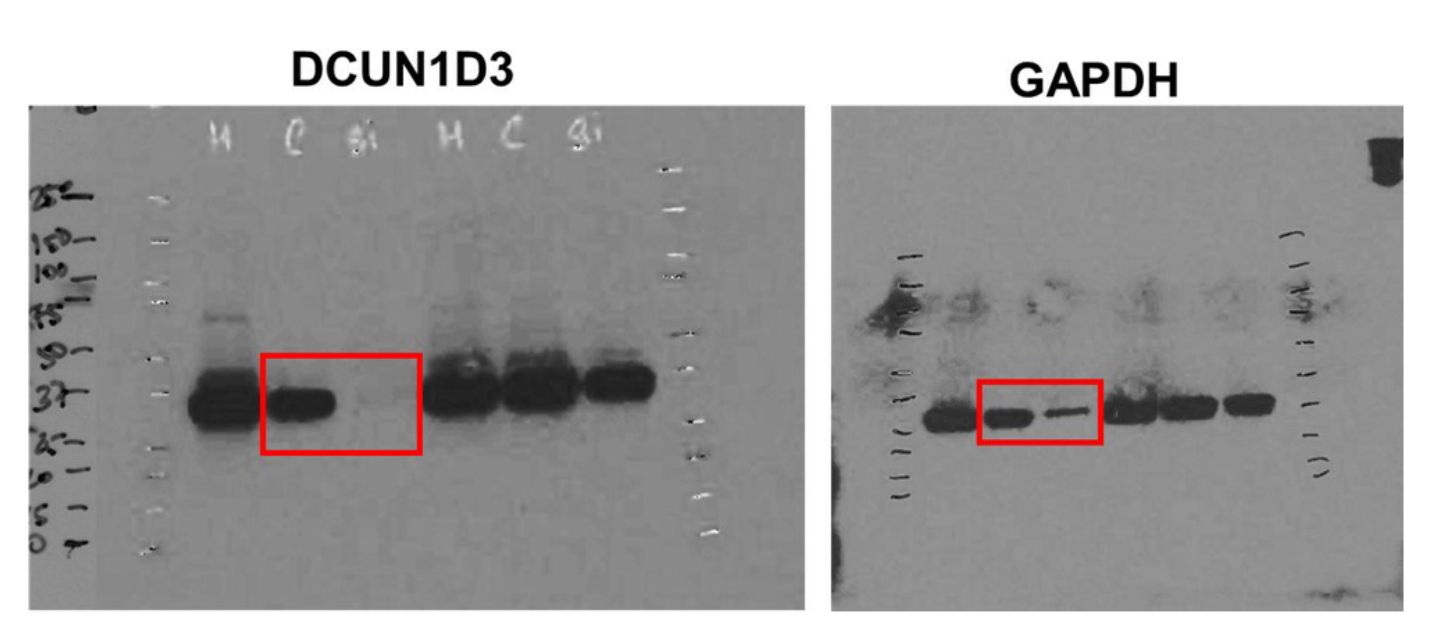
**

**Supplementary Figure S12.** Uncropped membrane stained with antibodies to DCUN1D3 and GAPDH.

**Supplementary Table 1: RNA-seq QC Parameters – Top Hat Alignment.**

| **Samples ID** | **Base quality (Q30 %)** | | **Number of reads** | | **Total Mapped Reads** | | **Uniquely mapped reads** | | **% Stranded** | | **Duplicate (%)** |
| --- | --- | --- | --- | --- | --- | --- | --- | --- | --- | --- | --- |
|  | **Read 1** | **Read 2** | **Read 1** | **Read 2** | **Read1** | **Read 2** | **Read 1** | **Read 2** | **Read 1** | **Read 2** |  |
| rs01 | 98 | 86 | 16041675 | 16041675 | 15720842 | 13795841 | 3696991 | 3665539 | 50.78 | 51.71 | 22.99 |
| rs02 | 54 | 49 | 8,589,812 | 8589812 | 4638498 | 4209008 | 544421 | 730941 | 49.49 | 45.64 | 21.84 |
| rs03 | 79 | 67 | 12768664 | 12768664 | 10087245 | 8555005 | 2498647 | 2643693 | 45.27 | 43.62 | 33.43 |
| rs04 | 60 | 50 | 9,736,983 | 9736983 | 5842190 | 4868492 | 2340660 | 2297828 | 45.02 | 42.34 | 35.68 |
| rs05 | 99 | 86 | 16035329 | 16035329 | 15874976 | 13790383 | 2429910 | 2630394 | 44.58 | 43.06 | 32.45 |
| rs06 | 81 | 74 | 13066077 | 13066077 | 10583522 | 9668897 | 229602 | 541468 | 49.68 | 43.09 | 16.28 |
| rs07 | 64 | 52 | 10362128 | 10362128 | 6631762 | 5388307 | 2571699 | 2524356 | 45.62 | 41.27 | 32.82 |
| rs08 | 54 | 45 | 8,947,352 | 8947352 | 4831570 | 4026308 | 2406341 | 2286649 | 49.58 | 45.84 | 26.91 |
| rs09 | 96 | 86 | 15614915 | 15614915 | 14990318 | 13428827 | 511360 | 1003763 | 49.20 | 47.55 | 28.15 |
| rs10 | 76 | 65 | 12798284 | 12798284 | 9726696 | 8318885 | 3072877 | 3038108 | 42.43 | 43.41 | 27.83 |
|  |  |  |  |  |  |  |  |  |  |  |  |
|  |  |  |  |  |  |  |  |  |  |  |  |
|  |  |  |  |  |  |  |  |  |  |  |  |

**Supplementary Table 2: Testing for residual effect from the crossover design (n=35).**

| **Measurements** | **Mean**  **(AS first)** | **SD**  **(AS first)** | **Mean**  **(SR first)** | **SD**  **(SR first)** | **P-value for residual effect** |
| --- | --- | --- | --- | --- | --- |
| **Oxidative stress (florescence intensity)** | 27.23 | 21.51 | 31.1 | 18.9 | 0.71 |
| **Catalase mRNA expression** | 0.91 | 1.27 | 1.07 | 0.95 | 0.63 |
| **SOD1 mRNA expression** | 0.65 | 0.72 | 1.37 | 2.03 | 0.12 |
| **Cullin3 florescence area (um^2^)** | 9.82 | 17.83 | 7.16 | 8.38 | 0.88 |
| **Nrf2-Cul3 co-localization florescence area (um^2^)** | 3.12 | 4.88 | 2.16 | 1.99 | 0.49 |
| **Nrf2 nuclear florescence area (um^2^)** | 1.69 | 3.87 | 1.68 | 1.82 | 0.29 |
| **DCUN1D3** | 0.5 | 1.3 | 0.74 | 1.32 | 0.45 |
| **SRF** | 0.76 | 0.79 | 0.74 | 0.78 | 0.97 |

Abbreviations: AS = adequate sleep; SR = sleep restriction; SOD1 = superoxide dismutase 1; Cul3 = cullin-3; Nrf2 = nuclear factor (erythroid-derived 2)-like 2; DCUN1D3 = defective in cullin neddylation 1 domain containing 3; SRF = serum response factor
